# Do educators value the promotion of students’ wellbeing? Quantifying educators’ attitudes toward wellbeing promotion

**DOI:** 10.1371/journal.pone.0273522

**Published:** 2022-08-29

**Authors:** David Byrne, Colm McGuinness, Aiden Carthy

**Affiliations:** Humanities Department, Technological University Dublin–Blanchardstown Campus, Dublin, Ireland; Yamaguchi University: Yamaguchi Daigaku, JAPAN

## Abstract

*Educators’ attitudes toward Social and Emotional Learning (SEL) and health and wellbeing promotion can significantly influence the success (or otherwise) of such policies and practices*. *While numerous studies exist*, *from which a broad understanding of such attitudes can be garnered*, *there is currently no attendant measurement which quantifies educators attitudes regarding the promotion of student wellbeing*. *The aim of this study was to address this gap in knowledge by quantifying the degree to which educators are positively or negatively disposed to the promotion of student wellbeing*. *The Attitudes Toward Wellbeing Promotion (ATWP) scale was administered to a diverse participant sample (n = 324)*, *which was opportunistically recruited from the population of post-primary educators in Ireland*. *Analyses using General Linear Modelling (GLM) identified several statistically significant differences in attitude*. *Main effects included position held in school and the presence/absence of practices such as streaming and vertical education*, while Interactions included educator gender*age and educator gender*single-sex/co-educational school status. *The results of this study suggest that*, *overall*, *educators appear to be positively disposed toward the promotion of student wellbeing*. *The highest levels of positivity were observed among female educators*, *particularly those working in all-girls schools*. *The lowest levels of positivity were observed among older male educators and educators working in schools that adopt streaming and vertical education practices*. This study provides the first baseline data pertaining to the degree to which post-primary educators may be positively or negatively disposed to the promotion of students’ social and emotional wellbeing.

## Introduction

Students’ social and emotional wellbeing is becoming an increasingly prominent consideration in educational contexts. Indeed, 2015 saw ‘wellbeing’ afforded formal recognition as an area of learning in post-primary schools in the Republic of Ireland [1]. This precipitated the development of a wellbeing curriculum, which consists of Social, Personal and Health Education (SPHE), Civic, Social and Political Education (CSPE) and Physical Education (PE). Schools are afforded a high degree of autonomy in developing and implementing appropriate wellbeing policies, delivering relevant wellbeing curricula, and realising appropriate whole-school wellbeing practices [2]. To facilitate schools in this endeavour, the National Council for Curriculum and Assessment (NCCA) in Ireland published the NCCA wellbeing guidelines in 2017. The stated aim of these guidelines is to “support schools in planning and developing a coherent wellbeing program that builds on the understandings, practices and curricula for wellbeing already existing in schools” [2 p8]. The guidelines are designed to support schools in actioning current wellbeing-related policies and practices and delivering the wellbeing curricula, with the aim of imbuing in students a wide range of cognitive, affective and behavioural skills that may augment their social and emotional wellbeing. The wellbeing guidelines came into effect in September 2017 and reified wellbeing as a whole-school endeavour. They also introduced the mandate that all post-primary schools in Ireland are to allocate 300 hours over the junior-cycle (the first three years of post-primary education) to the provision of social and emotional learning (SEL) and the promotion of students’ social and emotional wellbeing [3, 4].

It has been widely documented that large-scale ‘universal’ interventions that are designed to promote SEL and facilitate wellbeing-orientated policies and practices–such as the NCCA wellbeing guidelines–can be very beneficial in educational settings [4, 5]. Positive educator attitudes and perceptions have been found to be among the most influential factors in the success of such interventions and in achieving effective whole-school implementation of health- and wellbeing-orientated practices [6, 7]. Indeed, the NCCA highlight the importance of the educator in this regard, arguing that “wellbeing starts with the staff” [2 p29].

However, evidence from the literature suggests that, while educators are often able to identify the benefits of wellbeing promotion, their perceptions of SEL and health and wellbeing promotion may not always be positive [8, 9]. Indeed, Irish educators have previously been found to view wellbeing promotion in a negative light due to a perceived ubiquitous increase in workload that seems to be associated with increased efforts to promote student wellbeing [8]. Educators have expressed that a whole-school approach to wellbeing promotion adds to the already substantial pressure they feel in terms of delivering the core curriculum, both in terms of workload and the emotional labour associated with attending to their students’ wellbeing [8]. The stress of delivering the core curriculum can be so profound that, although educators can readily identify the merits of SEL and wellbeing promotion, they can become resentful of losing core curriculum activities at the expense of wellbeing curriculum activities [9]. In this regard, there is a distinct trend of the wellbeing curriculum and related activities being devalued in relation to the core curriculum and related activities [10]. This lack of parity afforded to the core and wellbeing curricula might also indicate structural shortcomings that further complicate educators’ perceptions of wellbeing promotion. For example, it has been found that as many as one third of SPHE educators across 394 participating schools lacked any kind of formal training in appropriate pedagogies for delivering this wellbeing curriculum subject [11]. Unsurprisingly, many educators have expressed discomfort with aspects of the wellbeing curriculum. More pointedly, some have been found to cede from delivery of some of the more challenging aspects of the curriculum–such as Relationships and Sexuality Education (RSE)–and instead emphasise other aspects of the wellbeing curriculum [7].

While evidence suggests that there are numerous factors that can negatively impact educators’ attitudes, it should be noted that these studies tend to suggest that educators do indeed identify the merits of wellbeing promotion [12, 13]. However, studies along these lines in Ireland have tended to focus on specific cohorts or specific aspects of the wellbeing curricula, and are often qualitative in nature [8, 14]. Studies that have employed quantitative methods generally focus on quantifying the number of educators who perceive wellbeing promotion a particular way or hold a particular attitude [7, 11]. As of yet, there is no attendant measurement of educators’ attitudes in this regard.

Developing a holistic understanding of educators’ attitudes regarding the promotion of student wellbeing would arguably be highly conducive to the success of related curricula, policies and practices [6, 7]. In this regard, there remains a significant gap in knowledge, in terms of our understanding of how much educators might value wellbeing promotion. The aim of the present study was to address this gap in knowledge by conducting a quantitative examination of the degree to which educators’ may be positively or negatively disposed to the promotion of student wellbeing. There were two research questions which this study aimed to address:

1. What are the attitudes and opinions of educators toward the promotion of Junior Cycle students’ wellbeing in Irish post-primary schools?
2. What are the attitudes and opinions of post-primary educators toward the current wellbeing guidelines published by the NCCA?

## Method

The data presented in this paper represent the quantitative phase of a larger, multi-methods study. This paper presents the results of the quantitative analysis of educators’ attitudes regarding the promotion of student wellbeing and the NCCA wellbeing guidelines. Ethical approval for this study was granted by the ethics committee at Technological University Dublin–Blanchardstown Campus. Written informed affirmative consent was obtained from participants for their anonymised responses to be used in this study and associated publications.

### Participants and settings

This study utilised an opportunistic sampling method [15]. A spreadsheet containing the names and contact information for 724 post-primary schools in the Republic of Ireland was obtained from the Department of Education and Skills (DES) [16]. Of these, 224 schools were used during the development of the Attitudes Toward Wellbeing Promotion (ATWP) scale [16], which was used in the present study. Participants were recruited from the remaining 500 unused schools on this DES contact list. Participation was open to any member of staff employed in a post-primary school in the Republic of Ireland. Exclusion criteria were established in relation to individuals that the TU Dublin ethics committee deemed to require special consideration within the present study, such as vulnerable individuals, minors, and educators employed in specialised fields, such as special needs and youth detention centres. A total of 327 educators (*n* = 117 males, *n* = 210 females) completed the survey. A crosstabulation of the age and gender of these participants can be seen in Table 1, while a crosstabulation of the participants’ employment context can be seen in Table 2.

**Table 1:** Crosstabulation of participants by age and gender.

		Gender		
		Male	Female	Total
Age	18–29	10	23	33
	30–39	28	62	90
	40–49	24	66	108
	50–59	33	51	84
	60+	4	8	12
Total		117	210	327

**Table 2:** Crosstabulation of participants by employment context.

			Urban/Rural		
SS_CoEd			Urban	Rural	Total
All boys	Position	Principal	0	0	0
		V. Principal	0	5	5
		Teacher	55	13	68
		Secretary/Admin.	0	0	0
		G. Counsellor	2	5	7
	Total		58	23	81
All girls	Position	Principal	2	2	4
		V. Principal	13	12	25
		Teacher	32	23	55
		Secretary/Admin.	1	0	1
		G. Counsellor	1	5	6
	Total		48	42	90
Co-education	Position	Principal	8	1	9
		V. Principal	5	8	13
		Teacher	0	0	0
		Secretary/Admin.	81	45	126
		G. Counsellor	4	4	8
	Total		98	58	156
Total	Position	Principal	10	3	13
		V. Principal	18	25	43
		Teacher	168	81	249
		Secretary/Admin.	1	0	1
		G. Counsellor	7	14	11
	Total		204	123	327

### Measures

The survey comprised four sections. Section one was a demographic questionnaire totalling 10 questions. Section two comprised the ATWP scale [17], and was aimed at directly addressing research question one. The ATWP scale is a 10 item, Likert response-format test instrument consisting of two non-correlated subscales:

- “Wellbeing Promotion” (Wel_Pro), which consists of five items and is designed to examine educators’ attitudes regarding the task of promoting student wellbeing.
- “Policies & Curriculum” (Pol_Cur), which consists of five items and is designed to examine educators’ attitudes regarding the available wellbeing policies and curricula.

The ATWP design is informed by the Affective, Behavioural, Cognitive (ABC) model of attitude [17] (see S1 Appendix for the composition of the ATWP). Each of the 10 items that comprise the ATWP produced item-Content Validity Index (i-CVI) values of 1.0, indicating the highest level of content validity. As such, the ATWP produced a scale-Content Validity Index (s-CVI) of 1.0. Internal reliability was interpreted as McDonald’s Omega (ω_t_). McDonald’s Omega was preferred to options such as Cronbach’s Alpha as Omega does not assume essential tau-equivalence and is therefore more suitable to reliability analysis of composite test instruments [18]. Reliability analysis indicated good reliability for the ATWP (ω_t_ = .81) and the Wellbeing Promotion subscale (ω_t_ = .82), and acceptable reliability for the Policies & Curriculum subscale (ω_t_ = .75).

Section three consisted of four non-composite Likert response format items pertaining to the NCCA wellbeing guidelines. These items were design to assess educators’ knowledge and use of the wellbeing guidelines, in addition to their perceptions of the usefulness of the guidelines. Finally, section four consisted of four open-ended questions intended to inform the design of the qualitative phase of this study (10). This analysis will not be discussed in the present paper.

### Procedure

Prior to dissemination to potential participants, the compiled survey was piloted with a small purposively sampled group of peers (n = 7). Feedback from the pilot study informed a number of minor corrections to the survey. The data obtained from the pilot study was not analysed as the purpose of this stage was to assess the functionality of the survey. A link to the survey, which was hosted online on Microsoft Forms, was then disseminated via email to the point of contact of 500 schools with the request that this link be forwarded to any members of staff who may be interested in participating in the research. The survey remained live for a period of two weeks. No attempts to garner additional participant numbers were made as a ‘sensitivity’ power analysis, conducted using G*Power (v3.1.9.4) indicated that the smallest detectable effect size was η_p_^2^ = .03, which was deemed acceptable for the present study. The average completion time for the survey was seven minutes and thirty-nine seconds.

### Analysis

Cleaning and screening of data resulted in three respondents being removed from the database on the basis of insufficiently completed responses (<50% completion) or providing patterned responses. As a result, 324 responses were taken forward for analysis. General Linear Models (GLMs) were used to explore the relationships between dependent variables (DVs) and independent variables (IVs). Data found to satisfy the assumptions of general linear modelling, as identified by [19]. All IVs were examined as main effects, while a number of interactions among IVs were also examined following evidence from available literature. For example, research has found that the attitudes of male and female *students* regarding their social and emotional wellbeing can vary across single-sex and co-educational settings [20, 21]. It was therefore undertaken to examine the attitudes of male and female *educators* toward wellbeing promotion in single-sex and co-educational settings. The model for each GLM consisted of eight IVs. Dichotomous IVs were ‘gender’, ‘urban/rural’, and ‘DEIS/non-DEIS’. Polytomous IVs were ‘age’, ‘position in school’, ‘number of students’, ‘single-sex/co-education’ (with single-sex split into ‘all-boys’ and ‘all-girls’ groups), and ‘use of streaming/vertical education’ (which also included use of ‘both’, ‘neither’ and ‘don’t know’ groups). Contrasts for main effects and interactions were calculated using estimated marginal means (EMMs), which indicate the mean response for each factor adjusted for any other variables in the model. Statistically significant main effects for polytomous IVs and interactions were subject to post-hoc testing using Fisher’s LSD test. Effect sizes for GLMs were interpreted as η_p_^2^. Effect sizes for Fishers LSD tests were interpreted as Cohen’s *d*, which was calculated as mean difference/sigma [22].

## Results

A GLM was run for each dependent variable in respect of the analytical model outlined in the analysis section. The GLMs for each of the four non-composite items pertaining to the NCCA wellbeing guidelines demonstrated extremely poor fit: NC1 –Familiar (*R*^*2*^ = .09, *p* = .52); NC2 –Knowledge (*R*^*2*^ = .08, *p* = .76); NC3 –Use (*R*^*2*^ = .12, *p* = .13), and; NC4 –Beneficial (*R*^*2*^ = .08, *p* = .75). Therefore, no further analyses were conducted for these models. Briefly, descriptive statistics for the non-composite items were as follows: NC1 –Familiar (*n* = 303, $\bar{x}$ = 3.50, SD = 1.90); NC2 –Knowledge (*n* = 305, $\bar{x}$ = 3.28, SD = 1.01); NC3 –Use (*n* = 304, $\bar{x}$ = 3.40, SD = 1.01, and; NC4 –Beneficial (*n* = 300, $\bar{x}$ = 3.63, SD = 10.5).

For clarity of reference, several variable labels have been abbreviated in results tables. Abbreviated variables are: Number of students (Num_students); Single-sex/Co-education (Single-sex/Co-Ed.); Streaming/Vertical education (Streaming/V. Education), and; Urban/Rural (Urb/Rur). Finally, the threshold between scores representing ‘negative’ and ‘positive’ attitudes was set at the median possible values, which, for ATWP was M = 30, and for Pol_Cur and Well_Pro were M = 15. Scores higher than these respective thresholds were considered to suggest positive attitudes, while scores lower than these thresholds were considered to suggest negative attitudes.

Results of analyses of variance for the ATWP, Well_Pro and Pol_Cur GLMs can be seen in S2–S4 Appendices, respectively. Post-hoc testing for each of the significant interactions and main effects was conducted as pairwise comparisons and will now be outlined.

### Gender by Age

Pairwise comparisons were conducted to examine for intra-gender mean differences across four age groups regarding the ATWP and the Pol_Cur subscale. Descriptive statistics can be seen in S5 Appendix. Pairwise comparisons regarding the ATWP identified a significant mean difference (mean diff. = 4.04) between male educators aged 30–39 and male educators aged 40–49. There was also a significant mean difference (mean diff. = 3.29) found between the 30–39 year-old and 50+ groups. This suggests that 30–39 year-old male educators held more positive overall attitudes regarding wellbeing promotion than did all of their older counterparts. Analyses also identified a significant mean difference (mean diff. = -2.67) between female educators aged 30–39 and female educators aged 40–49, as well as a mean difference of 2.30 between the 40–49 year-old and 50+ groups. This suggests that female educators aged 40–49 held more positive general attitudes regarding wellbeing promotion than their counterparts who were 30–39 years-old and those over the age of fifty.

As statistically significant differences were observed between 30–39 and 40–49 year-old age groups for both male and female educators, an interaction contrast was performed to compare the differences in these groups. A statistically significant difference between the differences was found, mean difference = 6.71, 95% CI [3.40, 10.02], *p* < .001, *d* = 1.32 (large). This suggests that an increase in age group from 30–39 to 40–49 was more profoundly negative regarding male educators’ ATWP scores than it was positive regarding female educators’ ATWP scores.

Inter-gender pairwise comparisons identified a significant mean difference (mean diff. -7.11) between male and female educators aged 40–49, as well as a mean difference of -4.06 between males and females in the 50+ age group. These findings suggest that male educators in both the 40–49 and 50+ age groups held less positive overall attitudes regarding wellbeing promotion than did their respective female counterparts. This was further analysed by performing an interaction contrast to compare the difference in male and female educators’ ATWP scores in the 40–49 year-old age group to the difference in male and female educators’ ATWP scores in the 50+ age group. A non-significant difference between the differences was found, mean difference = 3.05, 95% CI [-0.16, 6.26], *p* = .06.

Intra-gender pairwise comparisons were also conducted across the four age groups in relation to the Pol_Cur subscale. Analyses identified a statistically significant mean difference between male educators aged 30–39 and those in both the 40–49 age group, (mean diff. 2.58), and the 50+ age group, mean difference (1.92), suggesting that male educators aged 30–39 years-old held more positive attitudes regarding the available wellbeing policies and curricula than did their all of their older counterparts. Analyses also identified a statistically significant mean difference of -0.96 between female educators aged 30–39 and 40–49, as well as a statistically significant difference of 1.14 between female educators aged 40–49 and the 50+ age group. These findings suggest that female participants aged 40–49 years old held more positive attitudes regarding the available wellbeing policies and curricula than did their counterparts aged 30–39 and aged 50 and over.

As statistically significant differences were observed between 30–39 and 40–49 year-old age groups for both male and female educators, an interaction contrast was performed to compare the differences in these groups. A statistically significant difference between the differences was found, mean difference = 3.54, 95% CI [1.99, 5.09], *p* < .001. *d* = 1.45 (large). This suggests that an increase in age group from 30–39 to 40–49 was more profoundly negative regarding male educators’ Pol_Cur scores than it was positive regarding female educators’ Pol_Cur scores.

Pairwise comparisons were also conducted to examine for inter-gender differences across the different age groups. A statistical mean difference (mean diff. = -3.06) in Pol_Cur scores was identified between male and female educators aged 40–49 years-old, mean difference, suggesting that female educators aged 40–49 held more positive attitudes regarding the available wellbeing policies and curricula than did their male counterpart of a similar age. All statistically significant and non-significant test results for pairwise comparisons involving gender and age are displayed in Table 3.

**Table 3:** Pairwise comparisons of mean scores for “Gender by Age”.

							95% CI for Difference		
	Gender	Age		Mean Diff.	Std. Error	Sig.	Lower	Upper	d
ATWP	Male	18–29	30–39	-0.77	2.29	.74	-5.27	3.74	0.15
			40–49	3.28	2.13	.13	-0.93	7.48	0.65
			50+	2.52	2.21	.25	-1.83	6.88	0.50
		30–39	40–49	4.04**	1.38	.00	1.33	6.75	0.80
			50+	3.29*	1.45	.02	0.44	6.14	0.65
		40–49	50+	-0.75	1.27	.55	-3.25	1.75	0.15
	Female	18–29	30–39	2.69	1.40	.06	-0.07	5.45	0.53
			40–49	0.02	1.42	.99	-2.78	2.82	0.00
			50+	2.33	1.42	.10	-0.47	5.12	0.46
		30–39	40–49	-2.67*	0.96	.01	-4.55	-0.78	0.53
			50+	-0.36	0.99	.71	-2.31	1.58	0.07
		40–49	50+	2.30*	1.00	.02	0.34	4.27	0.45
Pol_Cur	Male	18–29	30–39	-0.84	1.08	.44	-2.97	1.28	0.35
			40–49	1.74	1.02	.09	-0.27	3.75	0.71
			50+	1.08	1.05	.30	-0.99	3.15	0.44
		30–39	40–49	2.58***	0.64	.00	1.33	3.83	1.06
			50+	1.92**	0.66	.00	0.62	3.23	0.79
		40–49	50+	-0.66	0.59	.27	-1.82	0.51	0.27
	Female	18–29	30–39	0.22	0.66	.74	-1.08	1.51	0.09
			40–49	-0.74	0.67	.27	-2.06	0.58	0.30
			50+	0.40	0.66	.55	-0.90	1.71	0.16
		30–39	40–49	-0.96	0.46	.04	-1.86	-0.06	0.39
			50+	0.19	0.46	.69	-0.72	1.09	0.08
		40–49	50+	1.14*	0.46	.01	0.24	2.05	0.47
	Age	Gender							
ATWP	18–29	Male	Female	-3.86	2.46	.12	-8.70	0.99	0.76
	30–39	Male	Female	-0.40	1.48	.79	-3.30	2.51	0.08
	40–49	Male	Female	-7.11***	1.45	.00	-9.96	-4.26	1.40
	50+	Male	Female	-4.06***	1.48	.00	-6.97	-1.14	0.80
Pol_Cur	18–29	Male	Female	-0.57	1.16	.62	-2.85	1.71	0.24
	30–39	Male	Female	0.48	0.69	.49	-0.88	1.85	0.20
	40–49	Male	Female	-3.06***	0.68	.00	-4.40	-1.71	1.26
	50+	Male	Female	-1.25	0.68	.07	-2.59	0.08	0.52

* p < .05

** p < .01

*** p < .001

### Gender by school type

Pairwise comparisons were conducted to examine for intra-gender differences across the three school types in relation to ATWP and the Well_Pro subscale. Descriptive statistics can be seen in S6 Appendix. Analyses regarding the ATWP identified a significant mean difference (mean diff. = 3.29) between male educators in all-boys schools and male educators in all-girls schools, suggesting that male educators in all-boys schools held more positive overall attitudes regarding wellbeing promotion than did their counterparts in all-girls schools. Pairwise comparisons also identified a significant mean difference of 2.94 between female educators in all-girls schools and co-educational schools, suggesting that female educators in all-girls schools held more positive overall attitudes regarding wellbeing promotion than did their counterparts in co-educational schools. Pairwise comparisons were also conducted to examine for inter-gender differences within each school type, with a significant inter-gender difference (mean diff. = -6.75) observed between male and female educators in all-girls schools, suggesting that, in all-girls schools, male educators held less positive overall attitudes regarding wellbeing promotion than did female educators.

Statistically significant intra- and inter-gender differences across the three school types were also observed in relation to the Well_Pro subscale. Analyses identified significant mean differences between male educators in all-boys schools and those in both all-girls schools (mean diff. = 1.84), and co-educational schools (mean diff. = 1.63). These findings suggest that male educators in all-boys schools held more positive attitudes regarding the task of promoting student wellbeing than did their counterparts in both all-girls and co-educational schools. There was also a significant mean difference (mean diff. = 1.83) between female educators in all-girls schools and co-educational schools, suggesting that female educators in all-girls schools held more positive attitudes regarding the task of promoting student wellbeing than did their counterparts in co-educational schools.

Pairwise comparisons also identified a significant mean difference between male and female educators in both all-girls schools (mean diff. = -3.90), and co-educational schools, (mean diff. = -1.87), suggesting that male educators held less positive attitudes regarding the task of promoting student wellbeing than female educators in both the all-girls and co-educational school setting. This was further analysed by performing an interaction contrast to compare the difference in male and female educators’ Well_Pro scores in the all-girls context to the difference in male and female educators’ Well_Pro scores in the co-education context. A statistically significant difference between the differences was found, mean difference = 2.03, 95% CI [0.22, 3.85], *p* = .03, *d* = 0.73 (medium). This suggests that, while female educators’ Well_Pro scores were higher than those of male educators in both contexts, the gender-difference in attitudes was more pronounced in all-girls schools. All significant and non-significant test results for pairwise comparisons involving gender and school type are displayed in Table 4.

**Table 4:** Pairwise comparisons of mean scores for “Gender by School Type”.

							95% CI for Difference		
	Gender	Single Sex/Co Ed.		Mean Diff.	Std. Error	Sig.	Lower	Upper	d
ATWP	Male	All boys	All girls	3.29*	1.48	.03	0.38	6.21	0.65
			Co-education	2.11	1.36	.12	-0.57	4.79	0.42
		All girls	Co-education	-1.18	1.57	.45	-4.28	1.91	0.23
	Female	All boys	All girls	-1.29	1.35	.34	-3.94	1.37	0.25
			Co-education	1.65	1.21	.17	-0.73	4.03	0.33
		All girls	Co-education	2.94*	1.06	.01	0.86	5.02	0.58
Well_Pro	Male	All boys	All girls	1.84*	0.80	.02	0.26	3.42	0.66
			Co-education	1.63*	0.73	.03	0.20	3.07	0.59
		All girls	Co-education	-0.21	0.86	.81	-1.89	1.48	0.07
	Female	All boys	All girls	-0.90	0.74	.22	-2.35	0.55	0.32
			Co-education	0.93	0.66	.16	-0.37	2.23	0.34
		All girls	Co-education	1.83**	0.58	.00	0.69	2.96	0.66
	Single Sex/Co Ed.	Gender							
	All boys	Male	Female	-2.18	1.65	.19	-5.43	1.08	0.43
	All girls	Male	Female	-6.75***	1.52	.00	-9.74	-3.77	1.33
	Co-education	Male	Female	-2.63	1.47	.07	-5.53	0.26	0.52
	All boys	Male	Female	-1.17	0.90	.19	-2.93	0.60	0.42
	All girls	Male	Female	-3.90***	0.80	.00	-5.48	-2.32	1.41
	Co-education	Male	Female	-1.87*	0.75	.01	-3.35	-0.39	0.68

* p < .05

** p < .01

*** p < .001

### Position

Pairwise comparisons were conducted to examine for mean differences among principals/vice principals, teachers and guidance counsellors in relation to the ATWP and each subscale (see S7 Appendix for descriptive statistics). Analyses regarding the ATWP identified a significant mean difference of 2.93 between principals/vice principals and teachers. This was reflected in relation to both subscales, with a statistically significant mean difference of 1.30 observed between principals/vice principals and teachers in relation to the Well_Pro subscale, and a mean difference of 1.38 in relation to the Pol_Cur subscale. A statistically significant difference (mean diff. = -1.58) was also noted between teachers and guidance counsellors. These findings suggest that teachers held less positive attitudes regarding the available wellbeing policies and curricula than did both principals/vice-principals and guidance counsellors. All statistically significant and non-significant test results for pairwise comparisons involving position are displayed in Table 5.

**Table 5:** Pairwise comparisons of mean scores for “Position”.

						95% CI for Difference		
	Position		Mean Diff.	Std. Error	Sig.	Lower	Upper	d
ATWP	Principal/V.Principal	Teacher	2.93**	0.91	.00	1.13	4.72	0.57
		Guidance Counsellor	0.58	1.58	.79	-2.54	3.70	0.11
	Teacher	Guidance Counsellor	-2.35	1.41	.10	-5.13	0.43	0.46
Pol_Cur	Principal/V.Principal	Teacher	1.38**	0.43	.00	0.53	2.23	0.57
		Guidance Counsellor	-0.20	0.74	.79	-1.65	1.25	0.08
	Teacher	Guidance Counsellor	-1.58*	0.66	.02	-2.88	-0.28	0.65
Well_Pro	Principal/V.Principal	Teacher	1.30*	0.50	.01	0.32	2.28	0.47
		Guidance Counsellor	1.01	0.84	.23	-0.64	2.65	0.36
	Teacher	Guidance Counsellor	-0.30	0.74	.69	-1.75	1.16	0.11

* p < .05

** p < .01

*** p < .001

### Streaming/Vertical education

Differences in attitudes were examined in relation to educational practices with regard to the ATWP and the Pol_Cur subscale. An interaction between educational practices and the urban/rural context was also examined in relation the Well_Pro subscale (see S8 Appendix for descriptive statistics). With regard to the ATWP, significant mean differences were found between the *both* group and the *streaming* group (mean diff. = -2.67), the *vertical education* group (mean diff = -4.69) and the *neither* group (mean diff. = -5.42). These findings suggest that educators in schools where *both* streaming and vertical education were employed held less positive overall attitudes regarding wellbeing promotion than did their counterparts in schools where *neither* or only one of these educational practices were employed. There were also significant mean differences between the *neither* group and the *streaming* group (mean diff. = 2.75), as well as the *don’t know* group (mean diff. = 4.78). Taken with the previous findings, this suggests that educators in schools where neither streaming nor vertical education are employed held more positive overall attitudes regarding wellbeing promotion than their counterparts in schools where streaming was employed, where educators are unsure if streaming or vertical education were employed, and where both streaming and vertical education were employed.

A significant mean difference was found between educators in the *vertical education* group and both the *streaming* group (mean diff. = 2.02), and the *don’t know* group (mean diff. = 4.04). Taken with the previous findings, this suggests that educators in schools where vertical education is employed held more positive overall attitudes regarding wellbeing promotion than their counterparts in schools where streaming was employed, where educators are unsure if either streaming or vertical education were employed, and where both streaming and vertical education were employed.

Statistically significant differences in attitude across educational practices were also observed in relation to the Pol_Cur subscale. Pairwise comparisons identified statistically significant mean differences between the *both* group and the *streaming* group (mean diff. = -1.23) the *vertical education* group (mean diff. = -1.68) and the *neither* group (mean diff. = -1.92). These findings suggest that educators in schools where both streaming and vertical education were employed held less positive attitudes regarding the available wellbeing policies and curricula than did their counterparts in schools where neither or only one of these educational practices were employed. There was also a statistically significant mean difference between the *neither* group and the *don’t know* group of 1.90, suggesting that educators in schools where neither streaming nor vertical education were employed held more positive attitudes regarding the available wellbeing policies and curricula than did their counterparts who were unsure if either of these educational practices were in play.

Finally, educational practices were found to interact with the school urban/rural status in relation to the Well_Pro subscale. Therefore, pairwise comparisons were conducted to examine for differences according to educational practice within both the urban and rural context. Analyses identified a significant mean difference of 2.11 between urban educators in the *neither* group and those in the *streaming* group, suggesting that, in the urban context, educators in schools where neither streaming nor vertical education were employed held more positive attitudes regarding the task of promoting student wellbeing that did their counterparts in schools where streaming was employed.

Within the rural context, significant mean differences were identified between educators in the *both* group and those in the *streaming* group (mean diff. = -2.59), the *vertical education group* (mean diff. = -3.92) and the *neither* group (mean diff. = -3.96). These findings suggest that, in the rural context, educators in schools where both streaming and vertical education were employed held less positive attitudes regarding the task of promoting student wellbeing than did their counterparts where only streaming, only vertical education, or neither of these educational practices were employed. A statistically significant mean difference was also identified between rural educators in the *neither* group and the *don’t know* group (mean diff. = 3.66), as well as between the *vertical education* group and the *don’t know* group (mean diff. = 3.61), suggesting that, in the rural context, educators in schools where neither streaming nor vertical education were employed, or where only vertical education was employed, held more positive attitudes regarding the task of promoting student wellbeing than did their counterparts who were unsure if either educational practice was in play.

Pairwise comparisons examining for differences between the urban and rural context according to educational practice identified a statistically significant mean difference between geographical contexts for the *both* group of 2.83, suggesting that, when schools practice both streaming and vertical education, urban educators held more positive attitudes regarding the task of promoting student wellbeing than did their rural counterparts. All statistically significant and non-significant test results for pairwise comparisons involving educational practice and school location are displayed in Table 6.

**Table 6:** Pairwise comparisons of mean scores for “Streaming/V.Education”.

							95% CI for Difference		
	Urban/Rural	Streaming/V. Education		Mean Diff.	Std. Error	Sig.	Lower	Upper	d
ATWP		Both	Streaming	-2.67*	1.16	.02	-4.94	-0.39	0.53
			Vertical education	-4.69***	1.18	.00	-7.00	-2.37	0.93
			Neither	-5.42***	1.12	.00	-7.62	-3.22	1.07
			Don’t know	-0.64	2.00	.75	-4.59	3.30	0.13
		Neither	Streaming	2.75**	0.88	.00	1.02	4.48	0.54
			Vertical education	0.73	0.83	.38	-0.91	2.37	0.14
			Don’t know	4.78**	1.84	.01	1.15	8.40	0.94
		V.Education	Streaming	2.02*	0.95	.03	0.16	3.88	0.40
			Don’t know	4.04*	1.86	.03	0.38	7.71	0.80
		Streaming	Don’t know	2.02	1.89	.28	-1.69	5.74	0.40
Pol_Cur		Both	Streaming	-1.23*	0.55	.03	-2.30	-0.15	0.50
			Vertical education	-1.68**	0.55	.00	-2.76	-0.59	0.69
			Neither	-1.92***	0.52	.00	-2.95	-0.89	0.79
			Don’t know	-0.03	0.95	.98	-1.91	1.85	0.01
		Neither	Streaming	0.70	0.41	.09	-0.12	1.51	0.29
			Vertical education	0.25	0.39	.53	-0.52	1.02	0.10
			Don’t know	1.90*	0.88	.03	0.16	3.63	0.78
		V.Education	Streaming	0.45	0.45	.32	-0.43	1.32	0.18
			Don’t know	1.65	0.89	.07	-0.11	3.40	0.68
		Streaming	Don’t know	1.20	0.91	.19	-0.58	2.98	0.49
Well_Pro	Urban	Both	Streaming	0.86	0.76	.26	-0.64	2.36	0.31
			Vertical education	-0.36	0.79	.65	-1.92	1.20	0.13
			Neither	-1.25	0.72	.09	-2.68	0.18	0.45
			Don’t know	-0.45	1.55	.77	-3.50	2.59	0.16
		Neither	Streaming	2.11***	0.58	.00	0.96	3.26	0.76
			Vertical education	0.89	0.57	.12	-0.24	2.01	0.32
			Don’t know	0.80	1.45	.58	-2.06	3.66	0.29
		V.Education	Streaming	1.22	0.65	.06	-0.06	2.51	0.44
			Don’t know	-0.09	1.49	.95	-3.02	2.85	0.03
		Streaming	Don’t know	-1.31	1.49	.38	-4.25	1.62	0.47
	Rural	Both	Streaming	-2.59*	1.03	.01	-4.62	-0.56	0.93
			Vertical education	-3.92***	0.98	.00	-5.85	-1.98	1.41
			Neither	-3.96***	0.96	.00	-5.84	-2.07	1.43
			Don’t know	-0.04	0.69	.95	-1.40	1.32	0.01
		Neither	Streaming	1.37	0.77	.08	-0.14	2.88	0.49
			Vertical education	0.04	0.69	.95	-1.32	1.40	0.01
			Don’t know	3.66*	1.38	.01	0.95	6.36	1.32
		V.Education	Streaming	1.33	0.79	.09	-0.23	2.88	0.48
			Don’t know	3.61*	1.38	.01	0.90	6.33	1.31
		Streaming	Don’t know	2.29	1.43	.11	-0.54	5.11	0.83
	Streaming/V. Education	Urban/Rural							
Well_Pro	Both	Urban	Rural	2.83*	1.04	.01	0.78	4.88	1.02
	Neither	Urban	Rural	0.13	0.57	.82	-1.00	1.25	0.05
	V. Education	Urban	Rural	-0.72	0.69	.30	-2.08	0.65	0.26
	Streaming	Urban	Rural	-0.61	0.78	.43	-2.15	0.92	0.22
	Don’t know	Urban	Rural	2.98	1.93	.12	-0.83	6.79	1.08

* p < .05

** p < .01

*** p < .001

## Discussion

### Gender by Age

Gender was constituent in two separate interactions with other variables, the first of which was age. Differences in attitudes appeared to be informed by considerations for the available wellbeing policies and curriculum, with no statistically significant differences observed in terms of attitudes pertaining to the task of promoting student wellbeing. Inter-gender differences were observed with regard to ATWP and Pol_Cur at the latter two age groups (50+ was non-significant for Pol_Cur). This seemed to be informed by a marked decrease in mean scores among male educators in the latter two age groups when compared to the two earliest age groups. Indeed, male educators aged 30–39 demonstrated statistically significantly more positive attitudes than did their counterparts aged 40–49, as well as those over 50 years of age. Additionally, the magnitude of the difference between the 18–29 year-old age group and each of the latter two age groups would suggest that these mean differences may be statistically significant if it were not for the small sample size of the 18–29 year-old age group.

Understanding how these differences may have manifested is rather difficult as there appears to be no available literature examining age differences in attitudes toward the promotion of student wellbeing, let alone interactions with gender. There are numerous studies examine age or experience in relation to factors that may be analogous to, or informative of, cognitive or affective aspects of educators’ attitudes [23–25]. However, these appear to be inconclusive in terms of explaining the gender and age differences noted in the present study. One particular line of inquiry that may provide factors that do in fact vary with regard to age or gender is research regarding personality.

Research regarding the Big Five model of personality has noted a strong tendency for different aspects of personality to vary by age and gender. What may be of particular interest in relation to the findings of the present study is the consistent finding that, generally speaking, openness to new experiences tends to decline at older age [26–28]. In fact, some studies have observed a curvilinear pattern in this regard, with openness increasing until middle-age (roughly 40–50 years of age) and then declining into older age [27, 29]. Furthermore, women tend to present as more open to new experiences than do men [29, 30], and it has been suggested that the decrease in openness in later years can be more sharply expressed among men than women [29]. This may be reflected in the present study, as both inter- and intra-gender differences in ATWP and Pol_Cur mean scores were largely influenced by a marked decrease in positivity among older male participants. As such, it may be the case that older male educators are less open to recent policies advancing the cause of wellbeing promotion and/or the recently codified wellbeing curriculum.

Openness may also go some way toward explaining the lack of such differences for Well_Pro mean scores. It could be argued that such differences may not manifest with regard to the task of promoting student wellbeing because educators have long understood the promotion of student wellbeing to be a fundamental task for which they are responsible [31]. Openness and attitudes regarding the task of promoting student wellbeing may therefore remain more stable across age. Conversely, formal recognition of wellbeing as an aspect of learning [1] precipitated changes to relevant policies and curricula, to which older educators (particularly male educators) may be less open and less positively disposed. Indeed, younger educators in Ireland have reported perceiving their older counterparts to be more ambivalent about, and disinterested in, aspects of the wellbeing curriculum [7]. Allying this to generally higher levels of reticence among male educators to become involved in wellbeing-related policies or practices [33] supports the proposition that openness may be influential in the observed differences in attitudes. However, this proposition does not explain the bimodal distribution of female mean scores across age groups for both ATWP and Pol_Cur. Female pre-service educators holding positive attitudes regarding aspects of the wellbeing curriculum [32] may explain the higher levels of positivity observed among 18–29 year old female participants. However, further research is recommended in order to understand the potential interaction between female gender and age with regard to the observed attitudinal differences.

### Gender by School type

The other statistically significant interaction observed with respect to gender involved the single-sex/co-educational status of participants’ respective schools. Attitudes regarding the available wellbeing policies and curricula did not appear to influence mean differences in ATWP scores. Rather, mean differences appeared to be informed by attitudes regarding the task of promoting student wellbeing. In this regard, female educators held more positive attitudes in both all-girls and co-educational schools than did male educators. This is somewhat congruent with previous research, as female educators have been found to advocate more strongly and optimistically for SEL and wellbeing promoting practices than have male educators [33]. Specific to the Irish context, female educators have been noted to be more receptive to both training and participation in SEL and wellbeing-orientated activities and practices [7], and to be more altruistically motivated to become involved in such [32]. The finding that the inter-gender difference is substantially larger in all-girls schools than in co-educational schools, and appears to be due to an increase in positivity among female educators in all-girls schools, suggests that all-girls schools are female educators’ preferred setting within which to attend to student wellbeing. Indeed, existing evidence suggests that female educators often tend to form closer and potentially more fulfilling relationships with female students than with male students [34, 35]. This could also be interpreted to indicate that female educators’ preferred student identity when attending to student wellbeing is feminine, thereby supporting the historical assertion that, generally speaking, educators’ preferred student identity is indeed feminine [36, 37].

The attitudes of male educators somewhat contrasted with those of female educators. Male educators indicated positive attitudes across the three school settings, but their mean ATWP and Well_Pro scores were markedly lower than were those of female educators, with the aforementioned statistically significant differences noted in the all-girls and co-education contexts. Socio-cultural factors may be implicit in explaining the differing attitudes toward the promotion of student wellbeing among male and female educators. Attitudes toward the provision of SEL or wellbeing-related activities may be subject to gender-norms that orient males toward stoicism, independence and introversion when addressing social or emotional health issues. Conversely, female educators may typically be oriented toward a sense of responsibility and caregiving in response to students’ displays of emotionality [33]. In Ireland, it has been noted that male educators are under-represented in SPHE service training [7] and, unsurprisingly, in the population of educators tasked with delivering SPHE [9]. The predominance of female educators delivering SPHE may be self-perpetuating, in that it may mean that female educators continue to be seen as carrying greater responsibility for delivering aspects of the wellbeing curriculum than male educators [7].

The potential absence of openness to a caregiving role among male educators may be problematic in terms of the long-term delivery of SPHE [9]. This issue may also be compounded by latent expectations of the role of male educators in school. Male educators can often prefer to adopt more dominant and authoritative approaches to their relationships with students [38]. The tacit expectation that male educators are good authority figures and effective disciplinarians [39] may further normalise them into these relationships, and away from the caregiving role. In this regard, it is not surprising to observe that male educators’ attitudes toward the task of promoting student wellbeing were more positive in all-boys schools than in both all-girls and co-educational schools.

Previous research has established that boys are often more disruptive, more conflictual, and pose greater challenges regarding classroom management than girls [34, 40, 41]. It has also been found that boys tend to become more aggressive in the all-boys context, while girls appear to have a ‘civilising effect’ upon boys in the co-educational context [40]. Male educators may see the potentially less hospitable environment of all-boys schools as a pertinent fit to their preference for dominant and authoritarian interpersonal relationships with students. For example, boys tend to present a greater challenge with regard to the delivery of some aspects of SPHE, notably RSE. However, evidence suggests that this challenge typically manifests as disruptive externalising behaviour, rather than issues pertaining to SEL or student wellbeing concerns [7]. On the other hand, girls can be more likely to seek the support of a caregiver in relation to their wellbeing needs [42]. In this regard, student gender presents an interesting caveat when examining gender differences in educators’ attendance to student wellbeing. While boys may be more challenging in terms of classroom management, it could be argued that male educators may feel vindicated in embracing a disciplinarian role in the all-boys context–perhaps ceding the caregiver role to female educators–and may feel they are less inclined to encounter students seeking the caregiver function from their teachers. This would be highly problematic, as it essentially constructs the all-boys context as a place where male educators may feel less obliged to attend to student wellbeing in an appropriate pastoral manner. This in turn would suggest that, contrary to historical assertions, the preferred student identity among male educators’ when attending to student wellbeing is in fact masculine.

### Position

Principals/vice-principals demonstrated more positive attitudes than did teachers across all three models, with all differences in attitudes being statistically significant. In addition, guidance counsellors were statistically significantly more positive regarding the available wellbeing policies and curriculum than were teachers. A small but non-significant difference in ATWP scores suggests that guidance counsellors were more positive about wellbeing promotion than were teachers. This may have been statistically significant but for the small size of the guidance counsellor group. Interestingly, there was also a small non-significant difference in Well_Pro scores that suggests principals/vice principals were more positive about the task of wellbeing promotion than were guidance counsellors. Overall, it appears that teachers tended to hold less positive attitudes, while principals’/vice-principals’ and guidance counsellors’ attitudes were comparable in most regards, with the (potential) notable exception of the task of promoting student wellbeing.

The extant literature suggests a number of explanations as to why teachers might present with less positive attitudes toward wellbeing promotion than principals/vice-principals or guidance counsellors. For example, it has been widely reported that an overcrowded curriculum or a perceived heavy workload can have a negative effect upon teachers’ perceptions of wellbeing-related policies, curricula and practices [9, 11, 43]. While teachers may see the value in caring for student wellbeing needs, when workload becomes overburdened in this way, SEL activities typically become de-prioritised in favour of academic activities. SEL activities are subsequently afforded less time in the school day [44]. Jourdan [45] went further to suggest that teachers may have a general tendency to prioritise the core curriculum and academic attainment over considerations for student wellbeing, while principals/vice-principals and guidance counsellors may be more cognisant of their responsibility regarding students’ wellbeing needs. Indeed, Irish post-primary teachers have been found to be somewhat resentful of losing their core curriculum subjects to facilitate wellbeing-related activities [9]. As such, teachers may feel more obliged toward core curricular tasks and may be less positively disposed toward SEL and the promotion of student health and wellbeing. Conversely, guidance counsellors in Irish post-primary schools have argued for their need to attend to the pastoral care of their students rather than being involved in core curricular activities [8, 46]. With this in mind, it could be argued that guidance counsellors’ low Well_Pro scores in the present study may reflect a reduced capacity to attend to their students’ wellbeing because of a requirement to undertake core curricular activities. Indeed, non-guidance related tasks have been identified as prominent predictors of negative affect and burnout among guidance counsellors [47].

The level of appropriate training received may also help to explain the differing levels of positivity demonstrated by participants. For example, a lack of appropriate training has been noted among Irish post-primary teachers who were tasked with SEL and health- and wellbeing-related activities [9, 32]. It has also been widely noted that a lack of appropriate training can result in negative perceptions of such activities [6, 9, 11, 32]. For example, insufficient training might inform a tendency for teachers to experience discomfort when attending to students’ SEL or health and wellbeing issues [7, 13], which in turn can be deleterious to teachers’ attitudes toward such responsibilities.

### Streaming/Vertical education

The extent to which schools employed streaming and/or vertical education was also found to be an important factor in this study. The findings suggest that the presence of both streaming and vertical education practices in participants’ schools is most deleterious to educators’ attitudes toward wellbeing promotion when compared to the presence of one or neither of these practices. Interestingly, the attitudes of participants in schools that employed vertical education were not statistically significantly different from those of the *neither* group, whereas participants in the *streaming* group demonstrated a statistically significantly lower level of positivity on the ATWP–and Well_Pro in the urban setting–than did the *neither* group. This would suggest that streaming may be the more influential factor in the consistently lower levels of positivity observed among *both* group participants.

Attempts to organise learning according to ability groupings in Ireland are well documented and the practice has also been widely debated in both the national and international literature [31, 48–50]. The effects of streaming upon Irish students are often negative, with students in lower stream classes tending to receive a higher number of negative teacher interactions. Interestingly, lower stream students also tend to receive a higher number of positive interactions [50]. However, negative interactions can be more efficacious in bringing about negative affect than positive interactions are at bringing about positive affect [51]. A particular source of anxiety for students is the potential for streaming to threaten friendships between students across different streams [31]. Evidence from the literature also suggests that a lower study culture, or even an anti-school counter-culture, can sometimes develop in lower stream classes [31, 48, 49]. This has previously been reflected in educators’ attitudes, with educators stating that students in lower streams can be more difficult to teach and can present a greater challenge with regard to classroom management [48, 49]. The findings of the present study appear to be congruent with the literature in this regard, as participants were found to hold less positive attitudes toward wellbeing promotion in contexts where streaming was utilised (i.e. in the *streaming* and *both* groups). While educators may perceive streaming as beneficial to the organisation of learning [48], it is possible that the increased difficulty of managing lower streams have a negative impact upon participants’ attitudes toward wellbeing promotion [50].

The findings indicate no statistically significant differences between the *neither* group and the *vertical education* group across the three models, suggesting that vertical education has little impact upon educators’ attitudes toward wellbeing promotion. Very little research into vertical education in post-primary schools currently exists. Vertical education appears to be more widely practiced in primary schools, and this is reflected in the quantity of availably literature. In primary schools, although educators can often be untrained in how to manage a multi-grade classroom or may incur a (perceived) heavier workload [52], educators’ attitudes toward vertical education are often positive.

The fact that, in the present study, *both* group mean scores were statistically significantly different from *streaming* group scores across all three models (a statistically significant difference was observed with regard to Well_Pro in the urban context) suggests that, while not an influential factor in and of itself, vertical education may be a compounding factor in the presence of other educational practices. Alternatively, it could be suggested that the presence of vertical education and/or streaming may be indicative of larger issues in relevant schools that may be deleterious to educators’ attitudes toward wellbeing promotion. Indeed, it has been widely argued that the adoption of such practices is rarely undertaken on the basis of pedagogic merit, but rather is precipitated by factors such as low pupil intake, a lack of staff or resources, or an over-emphasis of academic attainment [41, 48, 53]. As such, vertical education and/or streaming may not necessarily be the actual factors that influence participants’ attitudes, but rather may be indicative of wider structural or functional issues present in relevant schools. Indeed, insufficient staff and resources, and an over-emphasis upon academic attainment are strongly linked to poorer wellbeing outcomes for students and educators alike [9, 54–56]. Additional research would be necessary to further interpret the potential implications of these findings.

## Directions for future research

As previously mentioned, one of the strengths of this study is the gathering of novel data, which opens up a wide array of opportunities for future research. However, inherent in this strength is the limitation that practical and policy recommendations should arguably be withheld until further research is conducted, with a view to support or disconfirm the findings of the present study. Further research would be necessary to support the findings of this study before respective practice of policy recommendations could be considered to be evidence-based. It would also arguably be necessary to re-examine educators’ attitudes regarding the wellbeing guidelines, as analysis of differences in educators’ attitudes in this regard was inconclusive in this study.

If these findings were to be replicated, there are a number of areas that may benefit from additional research. For example, the interaction between gender and age could be more closely examined, with a view to exploring the hypothesis that older male educators might not fulfil the criteria of an ‘appropriate educator’ to deliver the wellbeing curriculum. It may also be fruitful to explore the interaction between gender and single-sex/co-educational contexts. In this regard, additional research could examine the dual hypotheses that, with regard to wellbeing promotion, male educators’ preferred student identity is masculine and female educators’ preferred student identity is feminine. Such a study could make a valuable contribution to the historical narrative that, generally speaking, educators’ preferred student identity is in fact feminine [36, 37]. Additional research may also shed further light upon the observed discrepancy in attitudes of principals/vice-principals and guidance counsellors and those of teachers. As previously discussed, there are a number of potential explanations as to why principals/vice-principals and guidance counsellors appeared more positive than teachers. Finally, it may be necessary to further examine the influence of non-wellbeing practices and policies upon best practice in wellbeing promotion, as these practices and policies were found to strongly and negatively affect educators’ attitudes toward wellbeing promotion in the present study.

## Limitations

There were some challenges with recruitment and sampling that should be considered. The sampling method used means it is not possible to ascertain an accurate response/non-response rate. There were also some limitations in terms of sample size. While the overall sample size was demonstrated to be appropriate via power analysis, there were a number of pairwise comparisons that presented with small samples in one or both groups (e.g. guidance counsellors or the 18–29 year old age group), which may indicate the potential occurrence of type ll errors [57]. Also, while the absence of an alpha correction was not necessarily a limitation, as this was a feature of the research design aimed at examining a broad array of areas for potential future research, the potential for type l errors should nevertheless be considered due to the large amount of tests run.

The analytical model demonstrated poor fit with regard to the ‘wellbeing guidelines’ Likert items. It is possible that poor fit may have been due, at least in part, to the design of these items, or educators lack of familiarity with the guidelines [10]. The reason for the poor fit is not inherently clear. However, the outcome is that, although overall attitudes were presented, the examination of variation in educators’ attitudes regarding the wellbeing guidelines was inconclusive.

Finally, while the use of a newly developed composite test instrument can be understood to be a strength in terms of the novel data that can be collected, it should be acknowledged that the novelty of this data presents a limitation with regard to the ability to contextualise and generalise the findings. While it is possible to assess the general positivity or negativity of educators observed in the present study against other studies, it is much more difficult to demonstrate support for the findings regarding the degree to which educators are positively or negatively disposed toward wellbeing promotion. As such, the ability to make practical or policy recommendations based on this single study is limited.

## Conclusion

This study provides the first baseline data pertaining to the degree to which post-primary educators may be positively or negatively disposed to the promotion of students’ social and emotional wellbeing. Overall, the findings of this study suggest that key stakeholders in this regard have demonstrated mostly positive attitudes. Recommendations have been made with regard to potential avenues of future research. The potential for future practice and policy recommendations resulting from the continuation of this line of research could be valuable in supporting all key stakeholders in achieving their highest potential as wellbeing educators, which would be crucially important as all educators are indeed wellbeing educators.

## Supporting information

S1 Data (CSV)

S1 Appendix (TIF)

S2 Appendix (TIF)

S3 Appendix (TIF)

S4 Appendix (TIF)

S5 Appendix (TIF)

S6 Appendix (TIF)

S7 Appendix (TIF)

S8 Appendix (TIF)
