# Supplementary figures and images for: Do educators value the promotion of students’ wellbeing? Quantifying educators’ attitudes toward wellbeing promotion
Source: PLoS One. 2022 Aug 29;17(8):e0273522. doi: 10.1371/journal.pone.0273522 (PMC9423639; doi:10.1371/journal.pone.0273522)

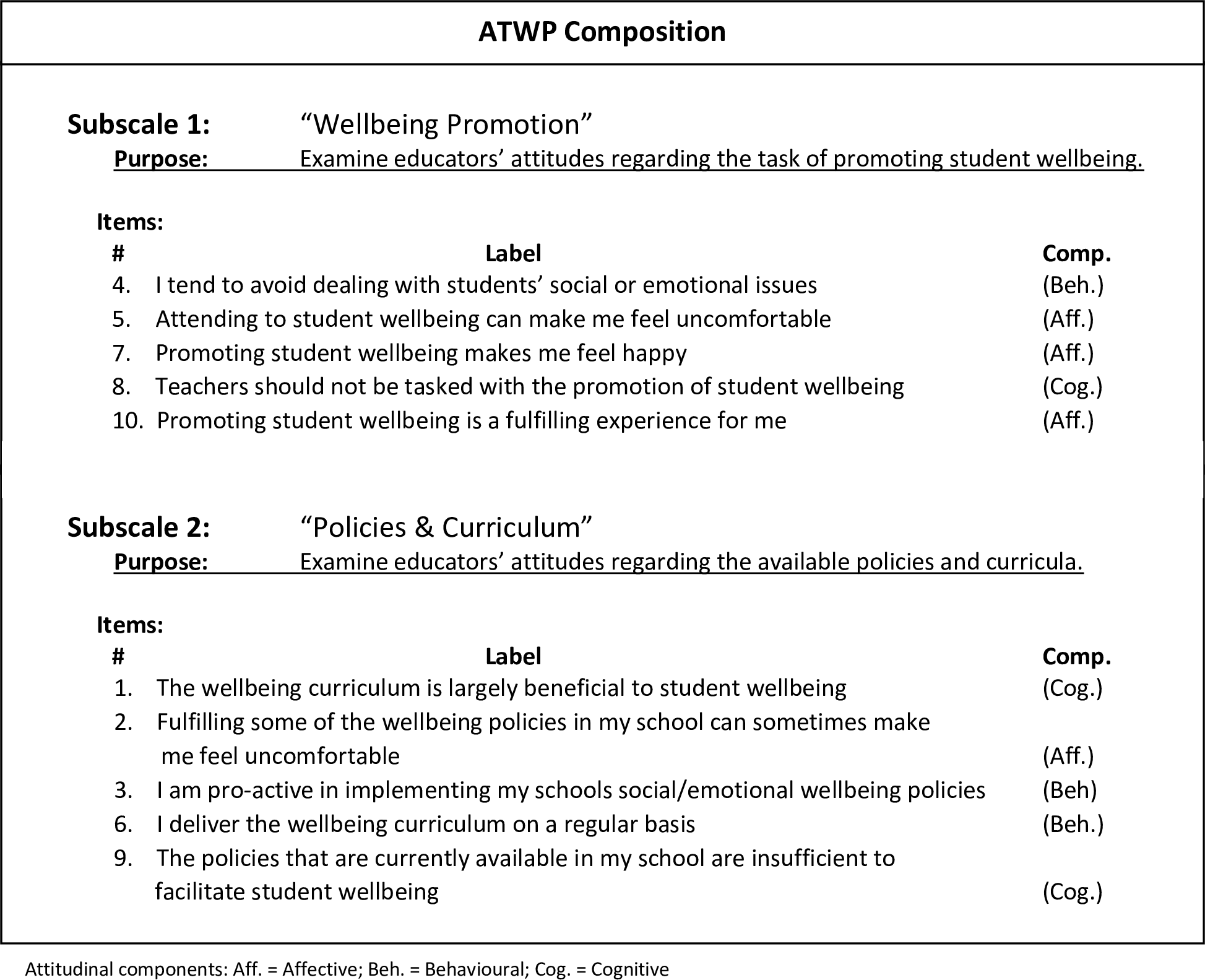

Supplement: S1 Appendix — (TIF) [file pone.0273522.s002.tif]

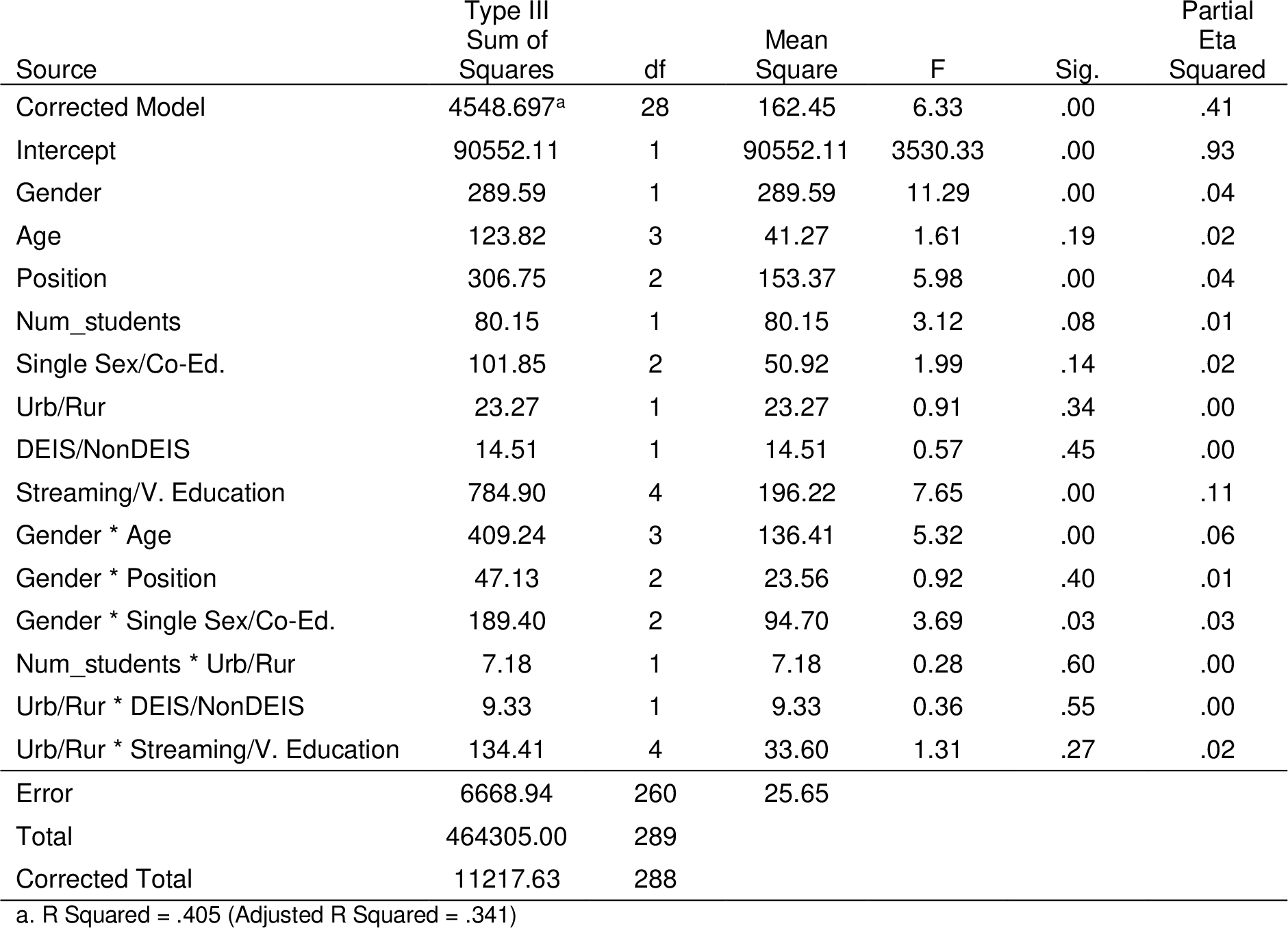

Supplement: S2 Appendix — (TIF) [file pone.0273522.s003.tif]

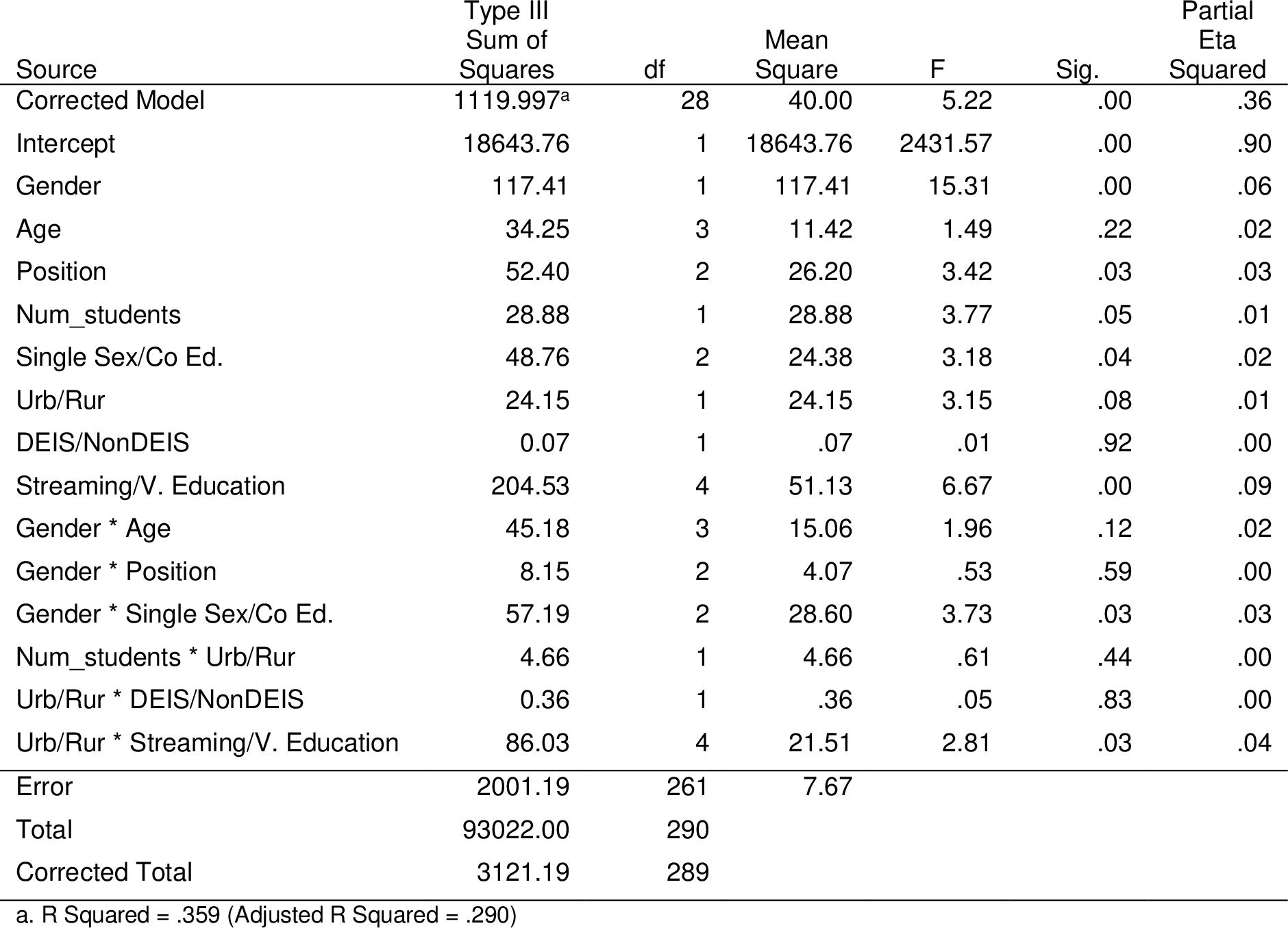

Supplement: S3 Appendix — (TIF) [file pone.0273522.s004.tif]

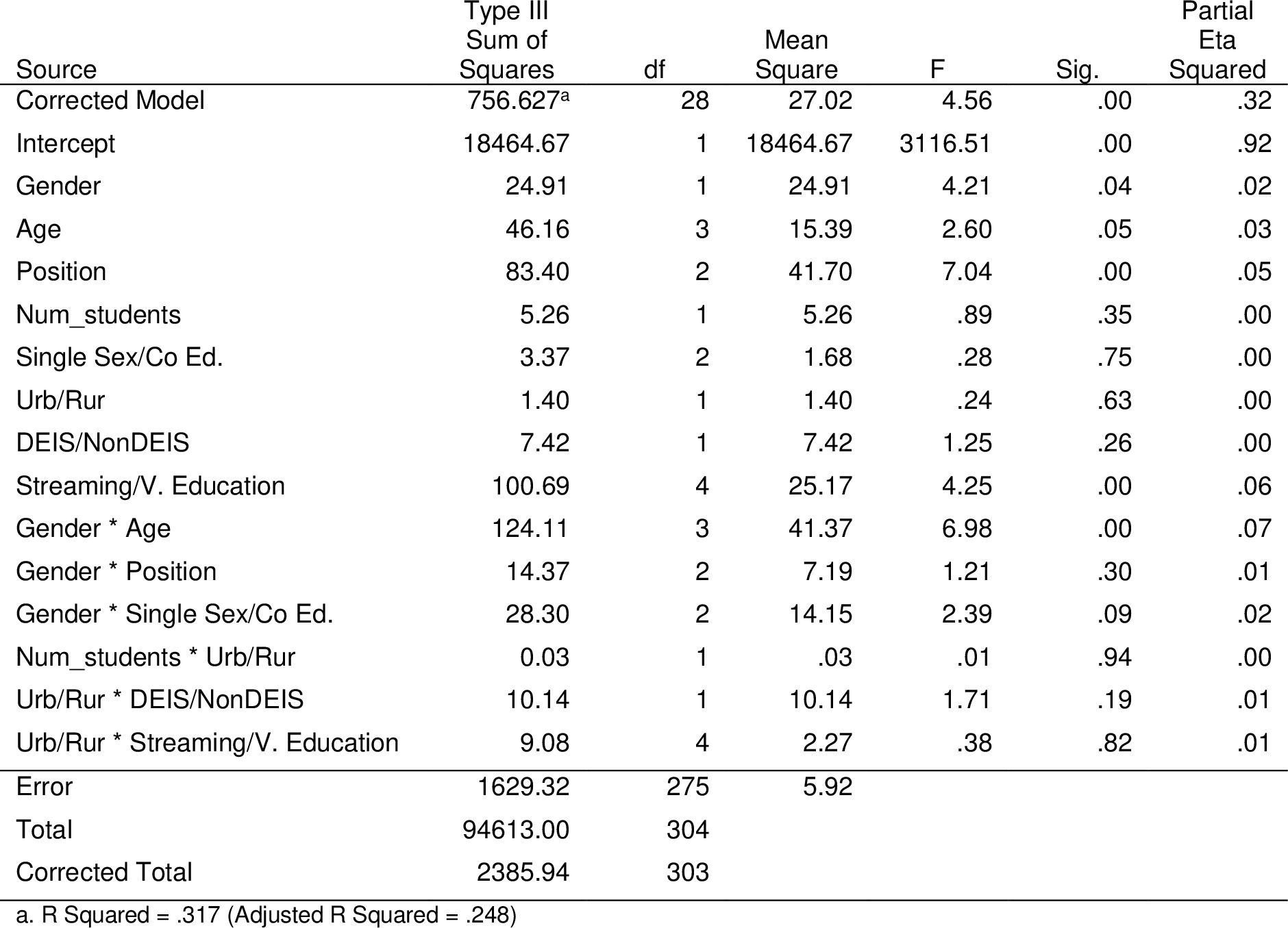

Supplement: S4 Appendix — (TIF) [file pone.0273522.s005.tif]

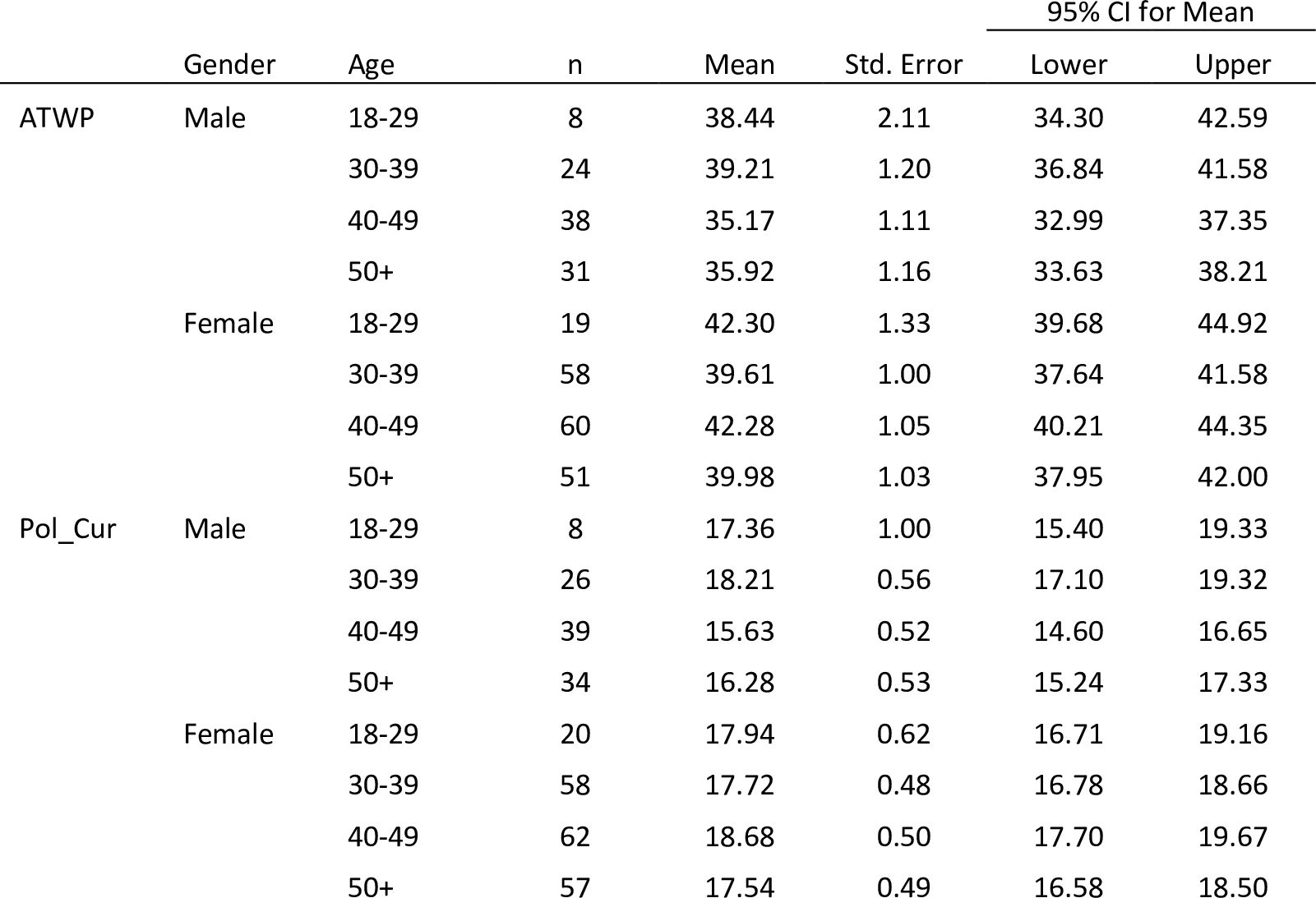

Supplement: S5 Appendix — (TIF) [file pone.0273522.s006.tif]

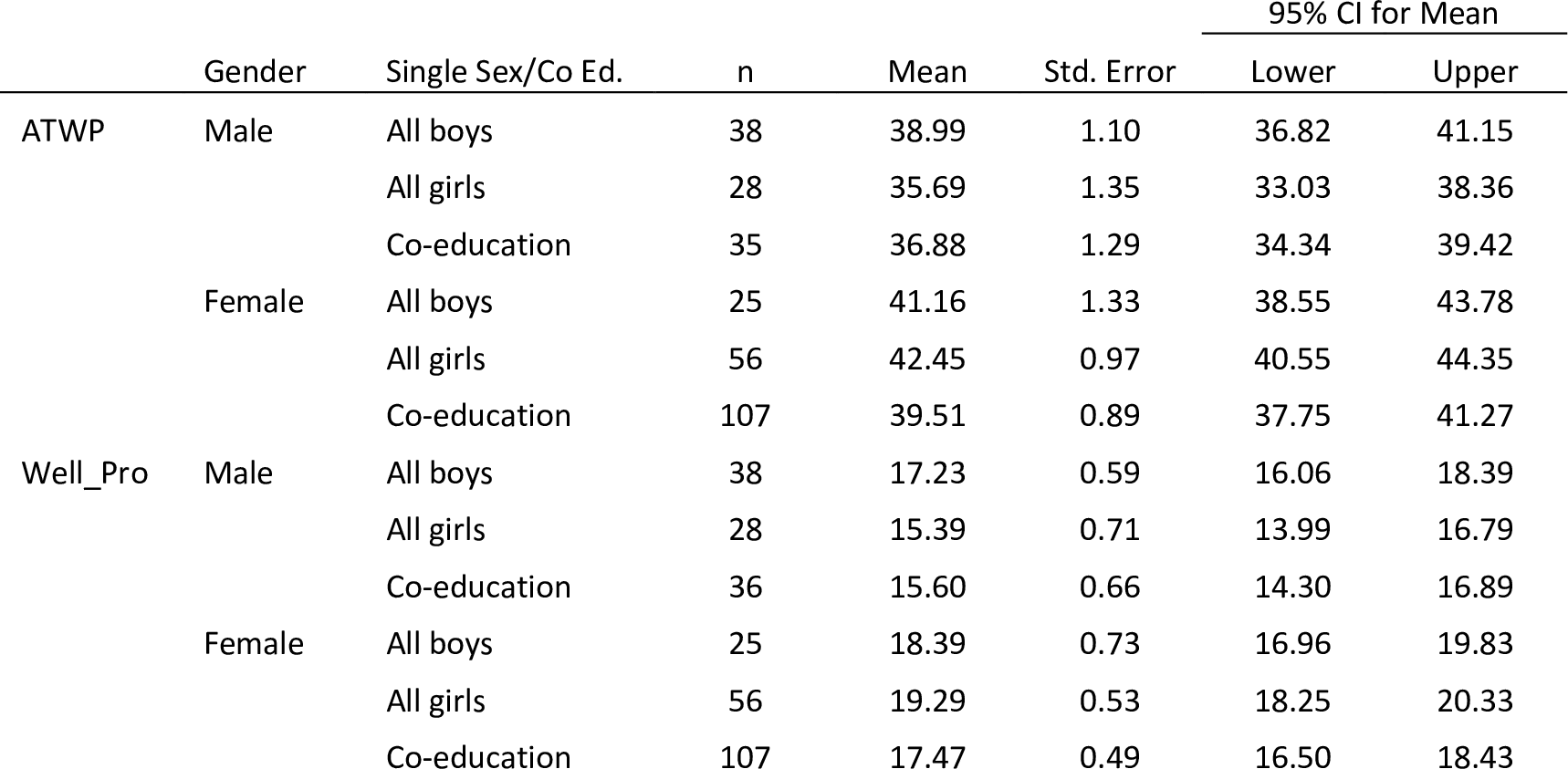

Supplement: S6 Appendix — (TIF) [file pone.0273522.s007.tif]

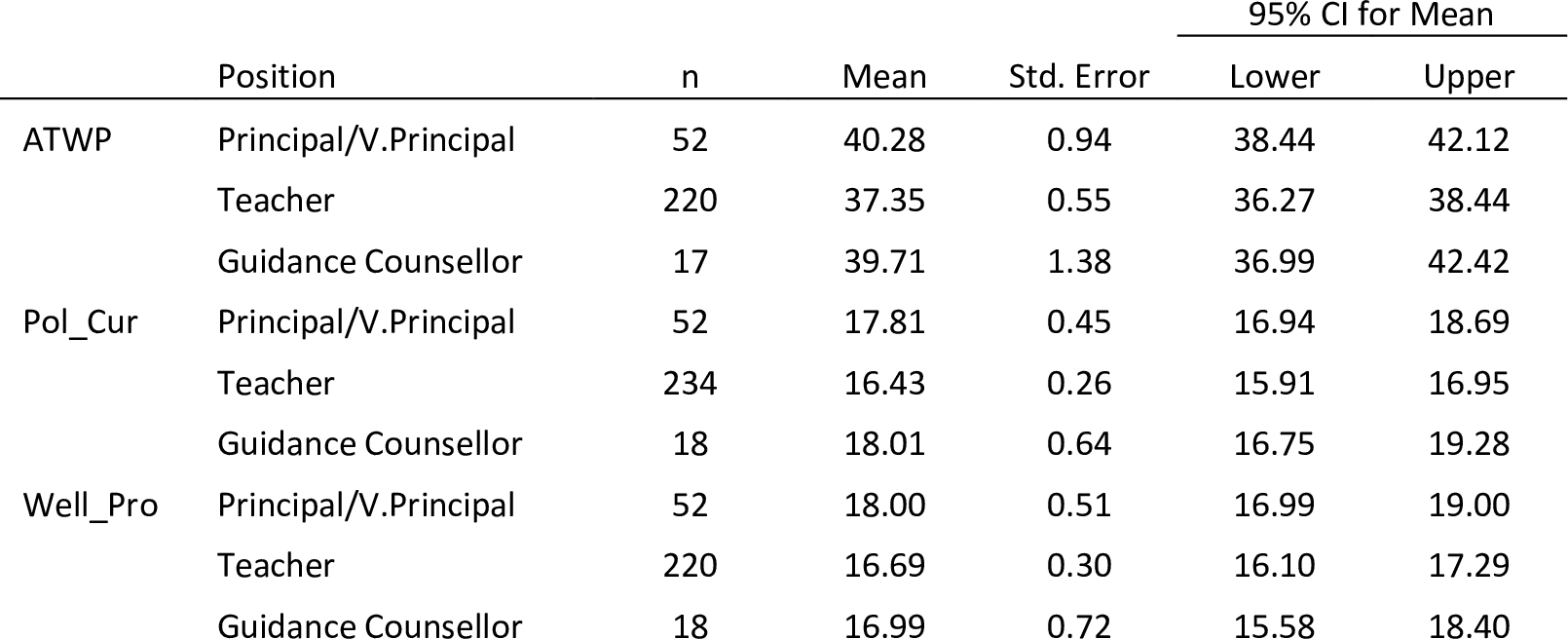

Supplement: S7 Appendix — (TIF) [file pone.0273522.s008.tif]

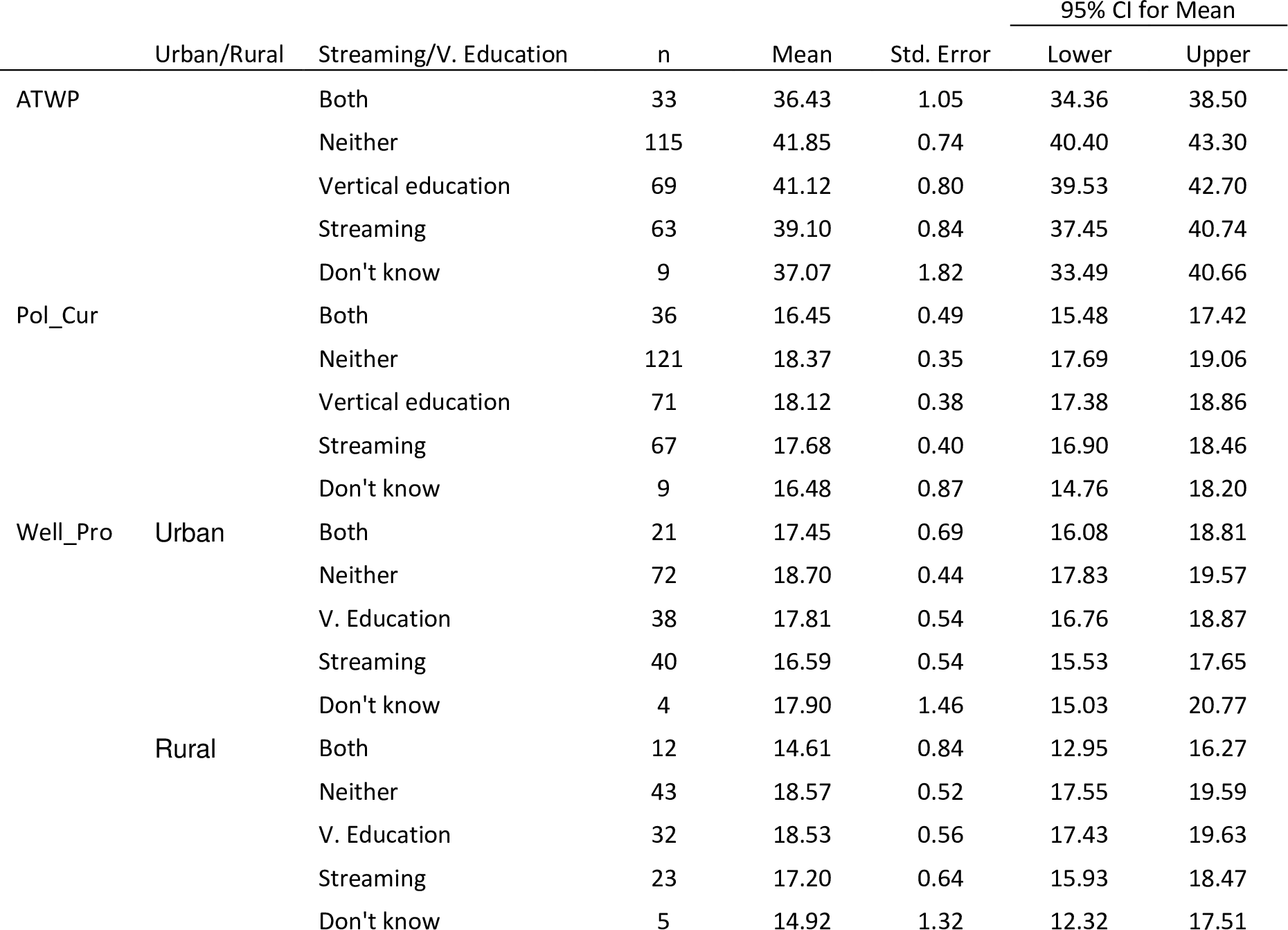

Supplement: S8 Appendix — (TIF) [file pone.0273522.s009.tif]
